# Alexithymia and Depression Affect Quality of Life in Patients With Chronic Pain: A Study on 205 Patients With Fibromyalgia

**DOI:** 10.3389/fpsyg.2018.00442

**Published:** 2018-04-04

**Authors:** Valentina Tesio, Marialaura Di Tella, Ada Ghiggia, Annunziata Romeo, Fabrizio Colonna, Enrico Fusaro, Giuliano C. Geminiani, Lorys Castelli

**Affiliations:** ^1^Department of Psychology, University of Turin, Turin, Italy; ^2^Clinical Psychology Unit, A.O.U. Città della Salute e della Scienza, Turin, Italy; ^3^Rheumatology Unit, A.O.U. Città della Salute e della Scienza, Turin, Italy

**Keywords:** fibromyalgia, alexithymia, depression, quality of life, chronic pain

## Abstract

Pain in fibromyalgia (FM) is accompanied by a heterogeneous series of other symptoms, which strongly affect patients’ quality of life and interfere with social and work performance. The present study aimed to evaluate the effects of alexithymia on both the physical and the psychosocial components of the health-related quality of life (HRQoL) of FM patients, controlling for the concomitant effects of depression, anxiety, and pain. In particular, given the strong interconnection between depression and alexithymia, the relationship between alexithymia and HRQoL as mediated by depressive symptoms was further investigated. Data were collected on a consecutive sample of 205 female patients with a main diagnosis of FM. The results showed that about 26% of the patients showed the presence of alexithymia, as assessed by the Toronto Alexithymia Scale (TAS-20). Clinically relevant levels of depressive and anxiety symptoms were present in 61 and 60% of the patients, respectively. The results of the hierarchical multiple regression analyses showed that pain intensity (PI) and depressive symptoms explained the 45% of the variance of the physical component of HRQoL (*p* < 0.001). Regarding the mental component of HRQoL, depressive and anxiety symptoms, alexithymia, and PI significantly explained 61% of the variance (*p* < 0.001). The mediation analyses confirmed that alexithymia had a direct effect on the mental component of HRQoL and showed a statistically significant indirect effect on both the physical and the mental components, through the mediation of depressive symptoms. In conclusion, the results of the present study suggested the presence of both a direct and an indirect effect of alexithymia, in particular of the difficulty identifying feeling, on the HRQoL of patients with FM. Indeed, even though the concomitant presence of depressive symptoms is responsible of an indirect effect, alexithymia *per se* seems to directly contribute to worsen the impact that this chronic pain pathology has on the patients’ quality of life, especially regarding the psychosocial functioning.

## Introduction

Fibromyalgia (FM) is characterized by widespread musculoskeletal pain, with palpation-specific regions of tenderness, associated with other symptoms, such as fatigue, non-restorative sleep, mood disorders, and cognitive impairment (Mease, 2005; Wolfe et al., 2010). This chronic pain syndrome has a complex and multifactorial etiopathogenesis and it affects mainly women (Williams and Gracely, 2007). Its prevalence ranges between 3 and 6% of the world population (World Health Organization (WHO), 2008), even though a recent literature review update showed a significant increase in FM prevalence worldwide (Marques et al., 2017). The heterogeneous series of physical and psychological symptoms experienced by FM patients has a negative impact on patients’ functioning and quality of life, strongly interfering with social and work performance (Sturge-Jacobs, 2002; Arnold et al., 2008). Among the psychological ones, depressive and anxiety symptoms have been widely reported, with prevalence ranging between 20 and 80% for depressive and between 13 and 64% for anxiety disorders (Bradley, 2005; Fietta et al., 2007).

Another psychological factor that has recently attracted the attention is alexithymia, a multifaceted personality dimension, largely observed in “psychosomatic” disorders (Taylor, 2000). Alexithymia has been related with several psychiatric and medical disorders, including chronic pain and FM (Lumley et al., 1997, 2002; Taylor et al., 1999; Celikel and Saatcioglu, 2006; van Leeuwen et al., 2012; Di Tella and Castelli, 2016). Alexithymia is characterized by a reduced ability to identify and describe subjective feelings, difficulty in distinguishing between feelings and bodily sensations of emotional arousal, restricted imagination processes, and a stimulus-bound, externally oriented cognitive style (Sifneos, 1973; Taylor et al., 1999). These deficits could interfere with the cognitive processing and regulation of emotions, resulting in increased negative affects (Martínez et al., 2015) and dysregulation of stress-related autonomic arousal, with chronic sympathetic hyperarousal (Lumley et al., 1996; Taylor and Bagby, 2012). In their study, indeed, Martínez et al. (2015) found that the difficulties in identifying their affective states, in interaction with negative pain appraisal (i.e., pain catastrophizing and fear of pain), could lead FM patients to develop emotional distress (in particular anxiety). Moreover, alexithymia can affect health perception, leading alexithymic subjects to misinterpret the bodily expressions of emotions as signs of physical disease and thus worsening the patients’ quality of life and enhancing the health care utilization (Lumley et al., 1996, 2007; Tuzer et al., 2011). Even though several studies have identified alexithymia as an important factor in FM syndrome (Sayar et al., 2004; Steinweg et al., 2011; Castelli et al., 2012; Di Tella et al., 2017; Ghiggia et al., 2017), the association between alexithymia and FM patients’ quality of life is not clear yet. The few evidences available suggest a possible role of negative affects, particularly depression, in mediating this relationship (Castelli et al., 2012). However, to the best of our knowledge, no study has deeply investigated yet the association between alexithymia and quality of life in FM patients.

The present study aimed thus to evaluate the effects of alexithymia on both the physical and the psychosocial components of the health-related quality of life (HRQoL) of FM patients, controlling for the concomitant effects of depression, anxiety, and pain. In particular, given the strong interconnection between depression and alexithymia, the relationship between alexithymia and HRQoL as mediated by depressive symptoms was further investigated. We hypothesized that the presence of alexithymic trait could negatively influence the impact that FM symptoms had on patients’ daily quality of life, both directly and indirectly.

## Materials and Methods

### Study Design

This prospective study was carried out on a consecutive series of FM patients recruited during their first visit at the Fibromyalgia Unit of the “A.O.U. Città della Salute e della Scienza – presidio Molinette” Hospital of Turin. The “A.O.U. Citta della Salute e della Scienza – A.O. Ordine Mauriziano of Turin – A.S.L. TO1 Ethic Committee” approved the study (“Psy-FM-AR Study,” procedure number CS/506) and all patients provided their written informed consent.

### Subjects and Procedure

At the end of their first visit at the Fibromyalgia Unit, all the female patients with a main diagnosis of FM, made by an expert rheumatologist, and fulfilling the inclusion/exclusion criteria were invited to participate. The main exclusion criteria were: less than 18 years old; low educational level (<5 years) or insufficient knowledge of the Italian language; unstable medical or psychiatric illness or current primary psychiatric diagnosis including severe depression; pain due to traumatic injury or structural/regional rheumatic disease. Out of the 354 consecutive FM patients contacted, 35 refused to participate in the study and 114 were excluded according to the exclusion criteria. A total of 205 patients composed the final sample. Recruited patients arranged an appointment with a trained doctoral clinical psychology student that assessed socio-demographic and clinical characteristics, and administered the pencil-and-paper psychological scales. The appointment lasted approximately 45 min.

### Variables and Instruments

#### Pain Characteristics

The pain intensity (PI) was measured using a Visual Analog Scale (VAS) and the Italian Pain Questionnaire (IPQ). Regarding the VAS, patients were asked to rate the average intensity of pain experienced in the last week on a scale ranging from 0 (No pain) to 10 (Extreme pain). In the IPQ (De Benedittis et al., 1988), a reconstructed Italian version of the McGill Pain Questionnaire, patients had to choose from 42 adjectives those that best described their pain. According to the model of Melzack and Torgerson (1971), the pain descriptors are divided into three main domains: sensory (IPQ-S), that measures modality and temporospatial qualities of pain, e.g., “stabbing,” “pulsing”; affective (IPQ-A), that measures tension, fear, and autonomic components of the pain experience, e.g., “nauseating,” “distressing”; and evaluative (IPQ-E) that measures the subjective intensity of the global pain experiences, e.g., “intolerable,” “annoying.” The sum scores of each of the dimensions of pain were reported as a portion (0–1) of the maximum possible score of each subscale.

#### Alexithymia

Alexithymia was assessed using the Italian version of the 20-Item Toronto Alexithymia Scale (TAS-20) (Bressi et al., 1996; Taylor and Bagby, 2004). The TAS-20 is composed by 20 items rated on a five-point Likert scale, from “strongly disagree” to “strongly agree.” In addition to the total score, the TAS-20 provides three subscale scores: “Difficulty identifying feelings” (DIF); “Difficulty describing feeling” (DDF); and “Externally oriented thinking scale” (EOT). According to the literature (Taylor et al., 1999), cut-off points were used to divide patients into non-alexithymic (total score ≤ 51), borderline (total score between 51 and 61), and alexithymic (total score ≥ 61).

#### Psychological Distress

The Italian version of the Hospital Anxiety and Depression Scale (HADS) (Costantini et al., 1999; Bjelland et al., 2002) was used to evaluate the presence of depressive and anxiety symptoms. It consists of 14 items divided into two subscales (range score: from 0 to 21): HADS-D for the depressive and HADS-A for the anxiety symptoms. A score of 8 or more suggests a clinically relevant level of depression/anxiety symptoms (Zigmond and Snaith, 1983).

#### Health-Related Quality of Life (HRQoL)

The Italian version of the Short-Form 36 Health Survey (SF-36) was used (McHorney et al., 1993; Apolone and Mosconi, 1998). It consists of 36 items divided into 8 subscales, which could be gathered into two main components: the Physical Component (SF-36_PC) composed by the Physical Functioning (PF), Physical Role Functioning (RP), Bodily Pain (BP), and General Health (GH) subscales; and the Mental Component (SF-36_MC) composed by the Vitality (VT), Social Functioning (SF), Emotional Role Functioning (RE), and Mental Health (MH) subscales. The scores range from 0 to 100, with the highest scores corresponding to the better condition.

### Statistical Analyses

Normal distribution was assessed following the criteria of absolute skewness (Sk) and kurtosis (K) values lower than 3.0 and 8.0, respectively (Kline, 2005). Based on these criteria, the assumption of normality was met for all the variables. Mean (SD) scores and frequencies were used as descriptive analyses, as appropriate. Pearson’s bivariate correlations were used to analyze the relationship between demographic, clinical and psychological variables, and HRQoL.

Two hierarchical multiple regression analyses were used to investigate whether alexithymia was a significant contributing factor for the explanation of the HRQoL in FM patients, using the Physical and Mental Components scores of the SF-36 as outcome variables. Stepwise method was used for variables inclusion of potentially confounding and competing predictors. To avoid unnecessary reductions in statistical power, confounding (age and educational level) and competing (depressive and anxiety symptoms, pain duration, and PI) predictors’ variables were included in the regression models only when they were significantly correlated with the outcome variables (*p*-value < 0.05). Collinearity was assessed using the statistical factors of tolerance and Variance Inflation Factor (VIF).

Lastly, a mediation analysis was conducted to test the mediating effect of depression on the association between alexithymia and patient’s HRQoL. As recommended by Preacher and Hayes (2004, 2008), mediation analysis procedures with bootstrap sampling were performed. The bootstrap method estimates indirect effects through one or more mediator variables with bias-corrected bootstrap confidence intervals (CIs) (Preacher and Hayes, 2004, 2008). A total of 1000 bootstrap resamples were used to generate bias-corrected 95% CIs for the indirect effect. Mediation is demonstrated when the indirect effect is significant and the CIs do not contain zero (Preacher and Hayes, 2004, 2008).

All the analyses were performed with the software “Statistical Package for Social Sciences – version 22” (SPSS-22). The mediation analyses were conducted using the PROCESS macro (Hayes, 2013), a computational procedure for SPSS.

## Results

Data on the socio-demographic and pain variables are reported in **Table 1**. Patients had a mean (SD) age of 51 (10) years and the majority of them had a secondary school degree at least. On average, the patients had chronic pain from 8 years and the mean PI was higher than 7. The result of the IPQ showed that patients reported the highest scores in the affective pain dimension (IPQ-A).

**Table 1:** Socio-demographic and clinical variables of the 205 patients.

		*N* (%)	Mean *(SD)*	Range
**Age**			51.84 (10.3)	24–74
**Educational level** (years)			10.78 (3.3)	5–18
	*Primary School*	*14* (*6.9*)		
	*Secondary School*	*83* (*41.1*)		
	*Higher School*	*90* (*44.6*)		
	*University*	*15* (*7.4*)		
**Marital status**				
	*Single*	*16* (*7.9*)		
	*Living together*	*14* (*6.9*)		
	*Married*	*138* (*68*)		
	*Divorced*	*26* (*12.8*)		
	*Widowed*	*9* (*4.4*)		
**Work status**				
	*Student*	*1* (*0.5*)		
	*Employed*	*116* (*57.7*)		
	*Unemployed*	*14* (*7*)		
	*Retired*	*32* (*15.9*)		
	*Housewife*	*38* (*18.9*)		
**Pain variables**				
	Pain duration (months)		102.11 (83.9)	4–420
	Pain intensity (VAS)		7.24 (2.4)	0–10
	IPQ		28.23 (13.4)	2–74
	*IPQ-S*		0.35 (0.15)	0–0.91
	*IPQ-A*		0.39 (0.21)	0–1
	*IPQ-E*		0.31 (0.21)	0–1


*IPQ (159 patients): Italian Pain Questionnaire; IPQ-S: Italian Pain Questionnaire-Sensory; IPQ-A: Italian Pain Questionnaire-Affective; IPQ-E: Italian Pain Questionnaire-Evaluative.*

Data regarding alexithymia and psychological distress are presented in **Table 2**. A total of 26% of the sample reported the presence of alexithymic trait at a clinical level (53 patients) and another 26% showed this trait at a subclinical level (54 patients). More than 60% of the patients reported a clinically relevant level of depressive/anxiety symptoms.

**Table 2:** Alexithymia, psychological distress, and health-related quality of life assessed with the Short-Form 36 Health Survey (SF-36).

	Mean *(SD)*	Range	*N* (%)
**TAS-20**	52.06 (13.2)		
*Non alexithymic (score* < *52)*			*95* (*47*)
*Borderline (score 52–60)*			*54* (*26.7*)
*Alexithymic (score* > *60)*			*53* (*26.2*)
DIF	20.45 (7.3)		
DDF	13.71 (4.9)		
EOT	17.9 (4.9)		
			
**HADS**	18.47 (8.1)		
HADS-D	9.18 (4.3)		*125* (*61.3*)*^#^*
HADS-A	9.29 (4.6)		*122* (*59.8*)*^#^*
			
**SF-36**			
**Physical Component**	**32.4 (15.7)**	**2.5–83.4**	
Physical Functioning	47.8 (21.4)	0–100	
Physical Role Functioning	17.3 (27.1)	0–100	
Bodily Pain	30.4 (16.8)	0–93.3	
General Health	34.3 (18.9)	0–90	
**Mental Component**	**39.6 (20.4)**	**6–94.3**	
Vitality	29.8 (17.5)	0–85	
Social Functioning	43.1 (22.2)	0–100	
Emotional Role Functioning	35 (39.7)	0–100	
Mental Health	50.6 (20.4)	0–96	
			


*TAS-20: Toronto Alexithymia Scale – total score; DIF: difficulty identifying feelings; DDF: difficulty describing feeling; EOT: externally oriented thinking scale; HADS-D/-A: Hospital Anxiety and Depression Scale-Depression/-Anxiety subscale; SF-36: Short-Form 36 Health Survey.*^#^*Number (%) of patients with a score above the cut-off (*score* ≥ *8*), suggesting the presence of a clinically relevant level of depressive/anxiety symptoms.*

Data regarding the HRQoL are summarized in **Table 2**. Patients reported very low mean values in all the subscales of the SF-36, suggesting the presence of a poor QoL in both its physical and mental components.

### Correlational Analyses

In order to verify possible relationships between clinical and psychological variables, and HRQoL, correlation analyses were performed (**Table 3**). Age, educational level, and pain duration were not significantly correlated with the physical and mental components of the SF-36. Pain was negatively and significantly correlated with both the SF-36_PC (*p* < 0.001) and the SF-36_MC (*p* < 0.001): the higher the PI, the lower the HRQoL. Statistically significant negative correlations were found between HADS-D and HADS-A, and both the physical and the mental components of the SF-36 (all *p* < 0.001), showing that higher psychological distress symptoms were correlated with a worse HRQoL. Regarding alexithymia, the DIF and DDF subscales of the TAS-20 were significantly correlated with both the SF-36_PC (DIF: *p* < 0.001; DDF: *p* = 0.006) and the SF-36_MC (both *p* < 0.001), suggesting that higher difficulty identifying and describing feelings were associated with a worse HRQoL. No significant correlations emerged between HRQoL and the EOT subscale of the TAS-20.

**Table 3:** Pearson’s correlations among socio-demographic, clinical, psychological distress, alexithymia, and quality of life assessed with the Short-Form 36 Health Survey (SF-36).

	1	2	3	4	5	6	7	8	9
1 – Age	–								
2 – Educational level	-0.218**	–							
3 – Pain Duration	0.255**	-0.018	–						
4 – VAS	-0.08	0.028	0.077	–					
5 – HADS-D	-0.012	-0.047	0.123	0.328**	–				
6 – HADS-A	-0.059	-0.019	0.137	0.362**	0.678**	–			
7 – TAS-20_DIF	-0.016	0.008	0.054	0.255**	0.526**	0.627**	–		
8 – TAS-20_DDF	0.097	-0.129	0.172*	0.231**	0.401**	0.463**	0.582**	–	
9 – TAS-20_EOT	0.106	-0.318**	0.058	0.051	0.170*	0.162*	0.202**	0.337**	–
10 – SF-36_PC	0.03	0.004	-0.126	-0.575**	-0.505**	-0.420**	-0.341**	-0.194**	-0.012
11 – SF-36_MC	0.058	-0.072	-0.042	-0.483**	-0.652**	-0.697**	-0.600**	-0.390**	-0.084


*VAS: Visual Analog Scale for pain intensity; HADS-D/-A: Hospital Anxiety and Depression Scale-Depression/-Anxiety subscale; TAS-20_DIF/_DDF/_EOT: Toronto Alexithymia Scale_Difficulty identifying feelings/Difficulty describing feeling/Externally oriented thinking subscale; SF-36_PC/_MC: Short-Form 36 Health Survey_Physical Component/Mental Component. ^∗^*p*-value < 0.05; ^∗∗^*p*-value < 0.01.*

Higher alexithymia scores were also significantly correlated with higher scores on the HADS-D (DIF: *p* < 0.001; DDF: *p* < 0.001; EOT: *p* = 0.016) and the HADS-A (DIF: *p* < 0.001; DDF: *p* < 0.001; EOT: *p* = 0.021) scales, suggesting that higher level of alexithymia were associated with a higher psychological distress. Both alexithymia (DIF: *p* < 0.001; DDF: *p* = 0.001) and psychological distress (HADS-D: *p* < 0.001; HADS-A: *p* < 0.001) were significantly correlated with the VAS score: the higher the PI, the higher the alexithymia and psychological distress symptoms.

### Regression Analyses

Two hierarchical multiple regression analyses were performed in order to investigate whether alexithymia was a significant predictor of both the physical and the mental components of HRQoL in FM patients. The variables age, educational level, pain duration, and the EOT subscale of the TAS-20 were no longer included in the regression analyses since they showed no significant correlation with the criterions. The DIF and the DDF subscales of the TAS-20 were, therefore, entered in the first regression block and the competing predictors [depression, anxiety, and PI (VAS)] were entered in the second block with the stepwise method.

The regression analysis regarding the Physical Component is reported in **Table 4**. In the first model, when only alexithymia features were entered into the analysis, the difficulty identifying feeling (DIF) subscale of the TAS-20 significantly predicted the criterion [β = -0.36, *t*(196) = -4.31, *p* < 0.001]. However, alexithymia ceased to be a predictive factor when depression was entered into the analysis, in the third model. The final model (model 3) explained a significant amount (45%) of the variance of the SF-36_PC [*F*(4,194) = 41.7, *p* < 0.001] and PI appeared to be the strongest contributor [β = -0.46, *t*(194) = -8.25, *p* < 0.001], followed by depression [β = -0.35, *t*(194) = -5.44, *p* < 0.001].

**Table 4:** Hierarchical multiple regression with Physical Component of Health-related Quality of life (SF-36_PC) as dependent variable (*N* = 199).

	Predictor	*R*^2^	Adj-*R*^2^	*F*	*F*-Δ*R*^2^	*B*	SE *B*	β	*p*
1		0.12	0.11	13.38^∗^	13.38^∗^				
	Constant					47.43	3.48		
	DIF					-0.78	0.18	-0.36	<0.001
	DDF					0.07	0.27	0.02	0.801
2		0.38	0.37	39.96^∗^	82.05^∗^				
	Constant					66.09	3.58		
	DIF					-0.54	0.15	-0.25	0.001
	DDF					0.24	0.23	0.07	0.299
	VAS					-3.29	0.35	-0.53	<0.001
3		0.46	0.45	41.74^∗^	29.54^∗^				
	Constant					66.75	3.35		
	DIF					-0.24	0.16	-0.11	0.129
	DDF					0.36	0.21	0.11	0.095
	VAS					-3.12	0.38	-0.46	<0.001
	HADS-D					-1.30	0.24	-0.35	<0.001


*^∗^*p*-value < 0.001. DIF: difficulty identifying feelings; DDF: difficulty describing feelings; HADS-D: Hospital Anxiety and Depression Scale-Depression; VAS: pain intensity.*

The regression analysis regarding the Mental Component is reported in **Table 5**. When only alexithymia features were entered into the analysis (model 1), the difficulty identifying feeling (DIF) subscale of the TAS-20 significantly predicted the criterion [β = -0.57, *t*(196) = -8.09, *p* < 0.001], with a 36% of the variance of the SF-36_MC explained. However, differently from the previous regression, alexithymia continued to be a predictive factor when competing predictors were entered into the analysis. The final model (model 4) explained a significant portion (61%) of the variance of the SF-36_MC [*F*(5,193) = 62.9, *p* < 0.001]. Anxiety symptoms [HADS-A: β = -0.30, *t*(193) = -4.39, *p* < 0.001], depressive symptoms [HADS-D: β = -0.25, *t*(193) = -4.13, *p* < 0.001] and alexithymia [DIF: β = -0.25, *t*(193) = -3.84, *p* < 0.001] followed by PI [VAS: β = -0.24, *t*(193) = -4.99, *p* < 0.001] were the factors that significantly predicted the SF-36_MC score.

**Table 5:** Hierarchical multiple regression with Mental Component of Quality of life (SF-36_MC) as dependent variable (*N* = 198).

	Predictor	*R*^2^	Adj-*R*^2^	*F*	*F*-Δ*R*^2^	*B*	SE *B*	β	*p*
1		0.37	0.36	56.45^∗^	56.45^∗^				
	Constant					75.22	3.80		<0.001
	DIF					-1.60	0.20	-0.57	<0.001
	DDF					-0.22	0.29	-0.05	0.457
2		0.53	0.52	72.80^∗^	67.31^∗^				
	Constant					76.38	3.29		<0.001
	DIF					-0.80	0.20	-0.29	<0.001
	DDF					0.07	0.26	0.02	0.788
	HADS-A					-2.31	0.28	-0.52	<0.001
3		0.59	0.58	68.74^∗^	27.22^∗^				
	Constant					87.96	3.81		<0.001
	DIF					-0.78	0.19	-0.28	<0.001
	DDF					0.13	0.24	0.03	0.600
	HADS-A					-1.92	0.28	-0.43	<0.001
	VAS					-2.25	0.43	-0.26	<0.001
4		0.62	0.61	62.95^∗^	17.03^∗^				
	Constant					89.62	3.68		<0.001
	DIF					-0.69	0.18	-0.25	<0.001
	DDF					0.18	0.23	0.04	0.444
	HADS-A					-1.32	0.30	-0.30	<0.001
	VAS					-2.08	0.42	-0.24	<0.001
	HADS-D					-1.22	0.30	-0.25	<0.001


*^∗^*p*-value < 0.001. DIF: difficulty identifying feelings; DDF: difficulty describing feelings; HADS-D/-A: Hospital Anxiety and Depression Scale-Depression/Anxiety; DT: Distress Thermometer; VAS: pain intensity.*

### Mediation Analysis

To deepen the results of the multiple regression analyses, a mediation analysis was conducted to test the relationship between alexithymia and the patients’ HRQoL mediated by depressive symptoms. On the basis of the results of the regression analyses, the DIF subscale was used.

**FIGURE 1:**
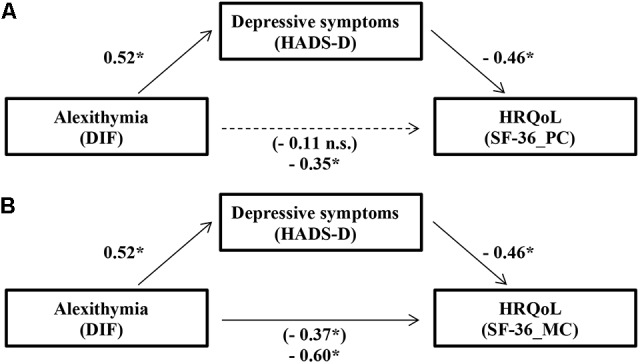
Standardized regression coefficients for the relationship between alexithymia and the Physical **(A)** and Mental **(B)** components of the SF-36 as mediated by depressive symptoms. The standardized regression coefficients between the difficulty identifying feeling (DIF) subscales of the TAS-20 and HRQoL, controlling for depressive symptoms, are in parentheses. ^∗^*p* < 0.001.

Regarding the Physical component (**Figure 1A**), the standardized regression coefficient between DIF and depressive symptoms was statistically significant (*p* < 0.001), as was the standardized regression coefficient between depressive symptoms and SF-36_PC (*p* < 0.001). The standardized indirect effect was (0.52)(-0.46) = -0.24. We tested the significance of this indirect effect using bootstrapping procedures. The bootstrapped unstandardized indirect effect was -0.52, and the 95% CI ranged from -0.73 to -0.35. Thus, the results confirm that, even if DIF did not have a direct effect on the SF-36_PC (standardized regression coefficient = -0.11, *p* = 0.13), it had a significant indirect effect (κ^2^ = 0.22) through depressive symptoms.

Concerning the Mental Component (**Figure 1B**), the standardized regression coefficient between depressive symptoms and SF-36_MC was statistically significant (*p* < 0.001). The standardized indirect effect was (0.52)(-0.46) = -0.24. The bootstrapped unstandardized indirect effect was -0.66, and the 95% CI ranged from -0.90 to -0.48. Thus, in addition to confirm the presence of a statistically significant direct effect (standardized regression coefficient = -0.37, *p* < 0.001), the results confirm that the difficulty identifying feeling subcomponent of alexithymia had a significant indirect effect on the Mental Component of the SF-36 (κ^2^ = 0.25) through depressive symptoms.

## Discussion

The present study evaluated the effects of alexithymia in a large sample of patients with FM, controlling for the concomitant effects of depression, anxiety, and pain. To deepen the investigation of the specific impact that alexithymia has on both the physical and the psychosocial components of the HRQoL, we also evaluated the mediation role of depressive symptoms. In fact, even though FM does not cause physical injuries or deformed joints, it causes a severe disability in daily living, negatively interfering with the patient’s functional, working, and psychosocial capacity (Sturge-Jacobs, 2002; Arnold et al., 2008). Available data underline that the negative impact on the quality of life may be even greater than those experienced in other chronic pain disorders and rheumatic conditions (Martinez et al., 1995; Bergman, 2005; Boonen et al., 2005; Birtane et al., 2007; Ubago Linares Mdel et al., 2008; White et al., 2008; Sicras-Mainar et al., 2009). In the current study, the results confirmed the great impact of the pathology on the patients’ quality of life, on both the physical and the mental components, with very low score in all the subscales of the SF-36.

Depressive disorders are the most frequent comorbid psychiatric conditions in FM with a prevalence of 20–80% (Fietta et al., 2007). In a community sample of 44,897 individuals, Kato et al. (2006) showed that 40% of patients with FM had current depressive symptoms without a formal diagnosis of depressive disorder whereas the rate of lifetime major depressive disorder comorbidity ranges from 62 to 86% (Aguglia et al., 2011). In line with these data, in the current study, the mean scores of both the depression and the anxiety subscales of the HADS were above the threshold, suggesting an extensive prevalence of clinically relevant depressive and anxiety symptoms, both present in about 60% of patients.

Up to date, studies evaluating the prevalence and the role of alexithymia in FM patients showed contrasting results (Di Tella and Castelli, 2013). Most studies agree on the higher prevalence of alexithymia in FM compared to healthy controls or to patients with other chronic pain pathologies such as rheumatoid arthritis (Sayar et al., 2004; Huber et al., 2009; Steinweg et al., 2011; Tuzer et al., 2011). The estimated prevalence of alexithymia in non-clinical samples ranges between 7 and 13% (Fukunishi et al., 1999; Mattila et al., 2006; Honkalampi et al., 2009), whereas according to the most recent reviews of the literature, the prevalence of alexithymia in patients with FM ranges from 15 to 20% (Di Tella and Castelli, 2013). Conforming to these data, 26% of the patients with FM recruited in the present study showed the presence of alexithymia and another 26% had a TAS-20 total score in the borderline range, suggesting the presence of a subclinical level of alexithymic traits. Some studies consider the high prevalence of alexithymia in FM as related to the concomitant high prevalence of depressive symptoms (Malt et al., 2002; Evren et al., 2006; Huber et al., 2009). Clinical researches conducted in various clinical settings have indeed shown a high incidence of alexithymia in patients with depression, which varies between 21 and 42% (Saarijärvi et al., 1993; Wise and Mann, 1995; Honkalampi et al., 2000, 2001; Taycan et al., 2017). Since both constructs share many characteristics, such as negative affect, decreased ability to communicate affect to other people, problems with interpersonal communication and less clarity about own feelings, it is reasonable to assume that there is an association between alexithymia and depression (Rude and McCarthy, 2003; Mattila et al., 2008; Picardi et al., 2011). Moreover, some researchers found that the decrease of the level of depression is associated with a TAS-20 scores decrease in patients with major depression, suggesting that alexithymia could be considered as a state-dependent phenomenon in people with depression (Honkalampi et al., 2000, 2001; Saarijärvi et al., 2001). Regarding chronic pain patients, previous studies, which evaluated alexithymia and depression in large heterogeneous chronic pain samples, suggested that depression worked as a full mediator between alexithymia and daily disability in FM (Makino et al., 2013; Saariaho et al., 2013; Shibata et al., 2014). Also the results of our previous exploratory study appeared to support this hypothesis (Castelli et al., 2012). In that study, even if the small sample size cautioned to interpret and generalize the data, the results seem to point out that the relationship between alexithymia and quality of life in FM could be totally mediated by the presence of psychological distress symptoms. In fact, alexithymia ceased to significantly contribute to the explanation of HRQoL when the psychological distress variables (depression for the physical component and anxiety for the mental component of HRQoL) were added as competing predictors (Castelli et al., 2012).

Given the exploratory nature and the small sample size of our previous study (Castelli et al., 2012), we performed a new study on a larger sample. The results of this last study confirmed the previous ones regarding the physical component of HRQoL. As in our previous work, alexithymia, in particular the difficulty identifying feeling subscale, ceased to significantly contribute to the explanation of the SF-36_PC when the depressive symptoms were added as competing predictor. PI and depression were the variables that significantly contributed to explain the variance of the SF-36_PC, with a good final model that explained 45% of the variance. Although PI and pain persistence in FM are independent from a coexisting depression or a concomitant psychological distress (Okifuji et al., 2000; Petzke et al., 2003), our results seem to suggest that these variables have a similar and additive effect in negatively influencing the physical functioning of FM patients, further supporting the multidimensionality nature of this pathology.

Regarding the psychosocial functioning, the results of the present study allowed us to go further with the results of our previous work (Castelli et al., 2012). Indeed, in the hierarchical multiple regression analysis performed on the mental component of HRQoL, alexithymia significantly contributed to the model even when controlling for the presence of psychological distress. Pain, anxiety and depressive symptoms, and the DIF subscale of the TAS-20 were all significant predictors of the SF-36_MC, with a final model explaining a very high percentage of the variance (61%).

This last result suggests that alexithymia has a significant direct effect on the psychosocial functioning of patients with FM. To better evaluate the weight of this effect on the mental component of the SF-36, and the presence of an indirect effect, mediated by depressive symptoms, on both the mental and the physical components, we performed the mediation analyses. The results further supported those of the regression model, confirming the presence of a significant direct effect of alexithymia on the mental component of the SF-36. These data provide methodologically grounded support to the hypothesis that the presence of alexithymia *per se* had a negative impact on FM patients’ HRQoL. Furthermore, the results showed that the DIF factor of the TAS-20 had a significant indirect effect on both the components of the HRQoL, mediated by depressive symptoms. The data showed, in fact, that the presence of difficulty identifying feelings had an important negative effect on the presence of depressive symptoms, which in turn negatively affects patients’ HRQoL. As showed by the κ^2^ value, the effect size of the indirect effect was medium for the physical component and large for the mental one. Taken together, these findings suggest that the patient difficulty identifying emotions may, on one hand, have an indirect effect, increasing symptoms of depression, which in turn may interfere with the individual’s ability to deal or cope with pain. On the other hand, the difficulty identifying emotions may also be considered a trait that directly influences illness behavior, as hypothesized by Lumley (Lumley et al., 1996). According to his model, the higher body awareness of individuals with alexithymia makes them focus on benign somatic sensations, thereby increasing sensation magnitude through a positive, autonomic feedback loop. As a result of the inability to identify accurately their own subjective feelings, they attribute these sensations to biological causes rather than psychological ones, thus experiencing these sensations as physical illness instead of the somatic manifestation of their own emotions (Lumley et al., 1996).

Such contrasting results regarding the relationship between alexithymia and depression in chronic pain could partially be the result of the negligence of the distinction between absolute and relative stability. Indeed, even if alexithymia lacks of absolute stability (i.e., its score may change in the presence of large changes in the severity of depressive symptoms), its relative stability has been demonstrated not only in patients with major depression, but also in patients with functional gastrointestinal disorders (Luminet et al., 2001; Porcelli et al., 2003; Taylor and Bagby, 2004). It can be argued that the lack of absolute stability of alexithymia could lead to attribute the high prevalence of alexithymia in FM only to the concomitant high prevalence of depressive symptoms, masking its direct effect in chronic pain patients, independently from the depressive symptoms.

Some main limitations have to be taken into account while considering the present study. Only female patients were evaluated and we did not include any control groups. Further studies comparing FM to other chronic pain pathologies should therefore be carried out. What is more, while this study contributes with important information regarding how alexithymia and depression may interact in FM, the cross-sectional nature of the study does not allow proving causal relationships. Future researches should address emotional deficits in this chronic pain pathology through longitudinal studies, assessing the variation of these co-occurring symptoms along the disease progression.

## Conclusion

The results of the present study suggest the presence of both a direct and an indirect effect of alexithymia on the HRQoL in patients with FM. Indeed, even though the concomitant presence of depressive symptoms is responsible of an indirect effect, alexithymia *per se* seems to directly contribute to worsen the impact that this chronic pain pathology has on the patients’ quality of life, especially regarding the psychosocial functioning. Taken together these results have an important implication for the treatment of FM patients. As recently reported, indeed, treatment strategies have on average only modest results in FM patients, calling for a more individualized management strategy (Castelnuovo et al., 2016a,b; Häuser et al., 2017). The presence of a direct and an indirect effect of alexithymia in FM, mediated by depressive symptoms, together with the evidence supporting the predictive role of alexithymia on the treatment outcome (Taylor and Bagby, 2004), further support the need for a multidimensional approach that includes its assessment. Even though there is an absence of established efficacious treatments for alexithymia, a recent review provides a strong case for the partial modifiability of alexithymia, but only by means of psychological interventions specifically intended to treat it (Cameron et al., 2014). According to the available research data, indeed, some aspects of alexithymia, as those of other dimensional personality traits, such as neuroticism or extraversion, may be more trait-like and thus enduring, whereas other aspects may be more state-dependent and thus changeable (Cameron et al., 2014). Furthermore, available research data suggest that identifying effective strategies for modifying alexithymia not only improves patients’ adaptive emotional processing, but also enhances other aspects of functioning (Cameron et al., 2014). Similar results were found in two very recent studies that evaluated the efficacy of an emotional awareness and expression therapy in chronic pain patients, including FM, which found that the decrease in alexithymia was linked to the improvements in PI and pain interference (Burger et al., 2016; Lumley et al., 2017). These data underline once again the importance to evaluate the presence of alexithymia in FM patients, in order to identify a patient-tailored therapy, aiming at optimizing treatment efficacy, and at minimizing costs and risks due to the use of ineffective therapies.

## Ethics Statement

This study was carried out in accordance with the recommendations of ‘Comitato Etico Interaziendale A.O.U. Città della Salute e della Scienza di Torino – A.O. Ordine Mauriziano – A.S.L. Città di Torino’ with written informed consent from all subjects. All subjects gave written informed consent in accordance with the Declaration of Helsinki. The protocol was approved by the ‘Comitato Etico Interaziendale A.O.U. Città della Salute e della Scienza di Torino – A.O. Ordine Mauriziano – A.S.L. Città di Torino.’

## Author Contributions

VT, LC, and GG were responsible for the conception and design of the study. AG, AR, FC, and EF were responsible for data collection and for clinical evaluations. VT and MDT were responsible for data analysis. LC and GG contributed to the interpretation of data. VT and MDT wrote the article, which was critically revised by all the other authors. All authors have approved the final version of the manuscript.

## Conflict of Interest Statement

The authors declare that the research was conducted in the absence of any commercial or financial relationships that could be construed as a potential conflict of interest.
